# Recurrent Loss of Macrodomain Activity in Host Immunity and Viral Proteins

**DOI:** 10.3390/pathogens12050674

**Published:** 2023-05-03

**Authors:** Sofia E. Delgado-Rodriguez, Andrew P. Ryan, Matthew D. Daugherty

**Affiliations:** Department of Molecular Biology, School of Biological Sciences, University of California—San Diego, La Jolla, CA 92093, USA

**Keywords:** ADP-ribosylation, macrodomain, PARP, host–virus evolution, phylogenetics, alphaviruses, coronaviruses

## Abstract

Protein post-translational modifications (PTMs) are an important battleground in the evolutionary arms races that are waged between the host innate immune system and viruses. One such PTM, ADP-ribosylation, has recently emerged as an important mediator of host antiviral immunity. Important for the host–virus conflict over this PTM is the addition of ADP-ribose by PARP proteins and removal of ADP-ribose by macrodomain-containing proteins. Interestingly, several host proteins, known as macroPARPs, contain macrodomains as well as a PARP domain, and these proteins are both important for the host antiviral immune response and evolving under very strong positive (diversifying) evolutionary selection. In addition, several viruses, including alphaviruses and coronaviruses, encode one or more macrodomains. Despite the presence of the conserved macrodomain fold, the enzymatic activity of many of these proteins has not been characterized. Here, we perform evolutionary and functional analyses to characterize the activity of macroPARP and viral macrodomains. We trace the evolutionary history of macroPARPs in metazoans and show that PARP9 and PARP14 contain a single active macrodomain, whereas PARP15 contains none. Interestingly, we also reveal several independent losses of macrodomain enzymatic activity within mammalian PARP14, including in the bat, ungulate, and carnivore lineages. Similar to macroPARPs, coronaviruses contain up to three macrodomains, with only the first displaying catalytic activity. Intriguingly, we also reveal the recurrent loss of macrodomain activity within the alphavirus group of viruses, including enzymatic loss in insect-specific alphaviruses as well as independent enzymatic losses in two human-infecting viruses. Together, our evolutionary and functional data reveal an unexpected turnover in macrodomain activity in both host antiviral proteins and viral proteins.

## 1. Introduction

ADP-ribosylation is a reversible post-translational modification (PTM) of proteins that is widely found in bacteria, eukaryotes, and viruses [1,2,3,4]. The PTM is catalyzed by diverse ADP-ribosyltransferases, including the family of PARP enzymes in eukaryotes [3]. Completing the cycle of PTM addition and removal, a variety of enzymatic domains can catalyze the removal of ADP-ribose from proteins [5]. Primary among these ADP-ribosylhydrolases is the macrodomain, which is a structurally conserved 120-200 amino acid module that can both recognize (‘read”) and reverse (‘erase”) ADP-ribosylation of proteins [5,6,7].

Macrodomains are found in a wide variety of eukaryotic, bacterial, and viral proteins. Of particular note are several metazoan proteins known as macroPARPs, which contain both a PARP domain (a “writer”) and two or more macrodomains (“readers” and “erasers”). Interestingly, mammalian macroPARPs, which include human PARP9, PARP14, and PARP15, are highly upregulated in response to the immune signaling molecule interferon (IFN), and have evolved under very strong positive (diversifying) selection [8], both of which are characteristic of genes that are engaged in host–pathogen evolutionary “arms races” [9,10,11]. Such data prompted us to propose that macroPARPs may be involved in a molecular and genetic conflict with viruses over ADP-ribosylation addition and removal [8]. Indeed, several papers have now revealed important roles for macroPARPs in directly or indirectly potentiating the host antiviral immune response, including evidence that PARP9 and PARP14 regulate the antiviral IFN response and other immune signaling pathways, and that PARP14 inhibits coronavirus replication [12,13,14,15,16]. However, the importance of macrodomains in these innate immune functions of macroPARPs has not been determined.

On the other side of the host–virus conflict surrounding ADP-ribosylation are viral proteins that contain macrodomains. Several groups of positive-sense single-stranded RNA (+ssRNA) viruses contain macrodomains embedded within non-structural proteins, including alphaviruses (e.g., chikungunya and equine encephalitis viruses), hepeviruses (e.g., hepatitis E virus), and coronaviruses (e.g., SARS-CoV-2) [17,18]. Notably, viral macrodomains have been shown to be critical for not only viral replication, but also for virulence and evasion of the IFN-mediated antiviral immune response [12,17,19,20,21,22,23,24,25,26]. In many cases, mutation of key catalytic residues in the viral macrodomain results in viral attenuation or increased sensitivity to antiviral immunity, suggesting that macrodomain ADP-ribosylhydrolase activity is a critical viral function.

These results position macrodomains and ADP-ribosylhydrolase activity at the center of a conflict between host antiviral immunity and viruses. As such, one expectation might be that macrodomain enzymatic activity would be well conserved throughout host and viral evolution. However, the degree to which macrodomains and ADP-ribosylhydrolase activity is conserved or divergent has not been analyzed in many cases. Here we analyze both host macroPARPs and viral macrodomain-containing proteins for conservation of key catalytic residues required for ADP-ribosylhydrolase activity. Strikingly, we find that key residues have been mutated in several independent lineages of host macroPARPs, as well as independent lineages of alphaviruses. Using an enzymatic assay for ADP-ribosylhydrolase activity in human cells, we confirm the loss of macrodomain activity consistent with the observed sequence changes. These results reveal at least three mammalian lineages in bats, ungulates, and carnivores that lack PARP14 macrodomain activity. Moreover, we find that macrodomains from several alphaviruses, including a human alphavirus and insect-specific alphaviruses, lack enzymatic activity. Together, our evolutionary and functional data reveal an unexpected turnover in macrodomain activity in both host antiviral proteins and viral proteins, shedding further light on the dynamic evolution of this critical PTM.

## 2. Materials and Methods

### 2.1. MacroPARP Homology Searches

A portion of human PARP14 (accession NP_060024.2) spanning the three macrodomains (residues 791-1387) was used to query the NCBI RefSeq protein database (including “metazoans (taxid:33208)”) using BLASTP [27] with an e-value cutoff of 1 × 10^−20^ and a query coverage cutoff of 40%. Using only the tandem macrodomain region as a search eliminated results from PARP proteins that lack macrodomains. The resulting 1846 sequences were downloaded as complete protein sequences and aligned using Clustal Omega [28]. Sequences that lacked a complete PARP domain were eliminated from further analyses, as were other incomplete sequences and poorly aligning proteins, resulting in 1091 “full length” macroPARP sequences. To eliminate closely related sequences and reduce total sequence number, sequences with >95% identity were reduced to a single unique sequence using CD-HIT with a 0.95 sequence identity cutoff [29]. The resulting 741 sequences are listed in Supplementary Material File S1. For genomes shown in Figure S1, the absence of PARP9 or PARP14 proteins was confirmed by performing a BLASTP search of the indicated genome with an e-value cutoff 0.05 and using the HMMER webserver [30] to search the indicated genomes with an e-value cutoff of 0.05. In all cases, and as expected, macrodomain-containing proteins were identified with these searches. However, all proteins that had both a macrodomain and a PARP domain that were identified in the *Petromyzon marinus*, *Asterias rubens*, *Crassostrea gigas*, and *Stylophora pistillata* genomes had a domain structure that resembled PARP14 rather than PARP9 or PARP15. Moreover, these searches yielded no protein in the *Drosophila melanogaster* and *Caenorhabditis elegans* genomes that contained both a macrodomain and a PARP domain, consistent with the conclusion that these genomes lack macroPARPs, based on previous iterative PSI-BLAST searches [31].

### 2.2. MacroPARP Phylogenetic Analyses

**Figure 1:**
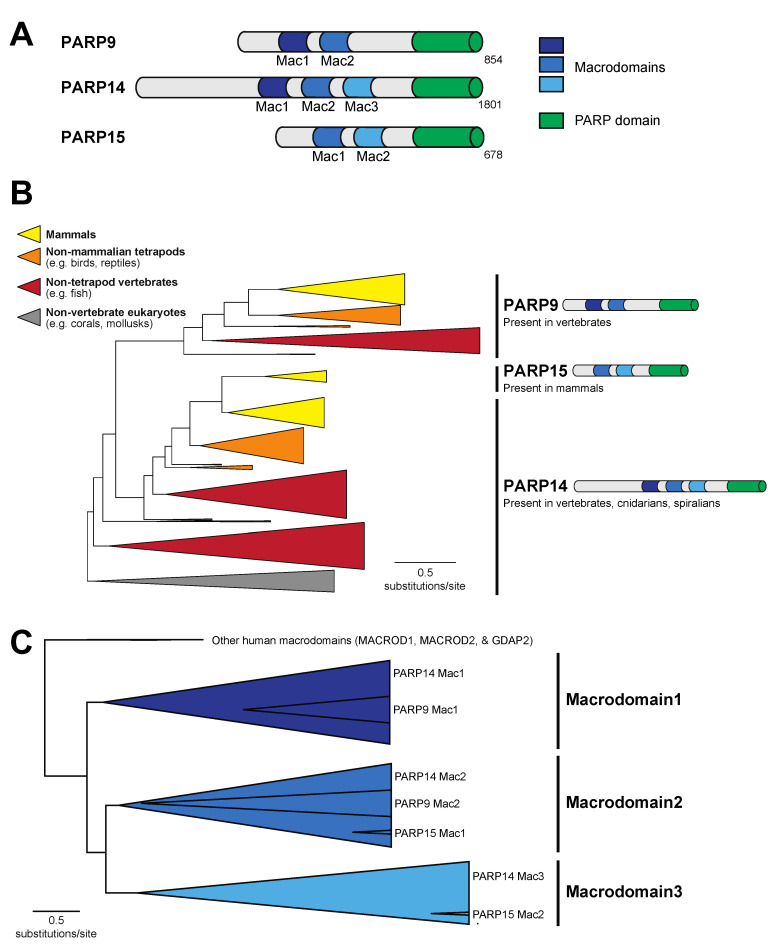
Evolution of macroPARP macrodomains within metazoans. (**A**) Domain structure of the three human macroPARP proteins, PARP9, PARP14, and PARP15. Macrodomains and PARP domains are shown, as is the total amino acid length of each protein. For simplicity, other domains within macroPARPs are not displayed. (**B**) Phylogenetic tree of metazoan macroPARP proteins. Clades of proteins with PARP9-like, PARP14-like, and PARP15-like domain architectures are indicated on the right. Colors represent groups of species as indicated in the key. (**C**) Phylogenetic tree of individual macroPARP macrodomains along with other indicated human macrodomains. There are three clear macroPARP macrodomain clades, corresponding to Macrodomains 1–3. As indicated, each large clade comprises two or three individual macrodomains from the metazoan macroPARPs.

All homologs shown in Supplemental File S1 were aligned using Clustal Omega using two iterations of refinement. For full-length macroPARP analyses, such as the one shown in Figure 1B, the alignment was trimmed to only the region spanning from the macrodomains to the PARP domain (corresponding to residues 791-1801 of human PARP14). Maximum likelihood phylogenetic trees were generated using IQ-TREE [32]. IQ-TREE phylogenies were generated using the “-bb 1000-alrt 1000” commands for generation of 1000 ultrafast bootstrap [33] and SH-aLRT support values. The best-fitting substitution model was determined by ModelFinder [34] using the “-m AUTO” command. For macrodomain analyses, such as the one shown in Figure 1C, the individual macrodomains were extracted from the full-length macroPARP alignment described above. Human PARP14 macrodomain boundaries were used: Macrodomain1–residues 791-978, Macrodomain1–residues 1003-1190, and Macrodomain3–residues 1216-1387. Individual extracted macrodomain alignments, along with macrodomains from human MACROD1 (accession NP_054786.2, residues 140-324), MACROD2 (accession NP_542407.2, residues 59-243), and GDAP2 (accession NP_060156.1, residues 43-226), were realigned using Clustal Omega with two rounds of refinement, and maximum likelihood phylogenetic trees were generated with IQ-TREE as described above. All phylogenetic trees were visualized using FigTree (http://tree.bio.ed.ac.uk/software/figtree/, accessed on 1 July 2022). All consensus logos were visualized using Geneious Prime 2022.1.1 (https://www.geneious.com/, accessed on 1 July 2022).

### 2.3. Coronavirus Macrodomain Homology Searches and Phylogenetic Analyses

The nsP3 protein from SARS-CoV-2 (accession YP_009724389.1) was used to query the NCBI RefSeq protein database (including “viruses (taxid:10239)”) using BLASTP with a query coverage cutoff of 25%. Resulting sequences were aligned and curated as for macroPARPs. Identical sequences were removed, but no CD-HIT removal of near-identical sequences was performed. Resulting sequences are listed in Supplemental File S2. Sequences were aligned using Clustal Omega with two rounds of refinement and maximum likelihood phylogenetic trees were generated using IQ-TREE.

### 2.4. Alphavirus Macrodomain Homology Searches and Phylogenetic Analyses

The non-structural polyprotein from Sindbis virus (accession NP_062888.1) was used to query the NCBI RefSeq protein database (including “viruses (taxid:10239)”) using BLASTP with a query coverage cutoff of 25%. Resulting sequences were aligned and curated as for macroPARPs. Identical sequences were removed, but no CD-HIT removal of near-identical sequences was performed. Resulting sequences are listed in Supplemental File S3. Sequences were aligned using Clustal Omega with two rounds of refinement and maximum likelihood phylogenetic trees were generated using IQ-TREE. 

### 2.5. SARS-CoV-2 Macrodomain2 and Macrodomain3 Structure Prediction

Sequences for Macrodomain2 (residues 415–541) and Macrodomain3 (residues 549–676) were extracted from the SARS-CoV-2 nsp3 region of the ORF1ab polyprotein (accession YP_009724389.1). Structural models for these domains were predicted using AlphaFold2 via the ColabFold package [35] with default parameters. Although the sequence similarity is low, there was a clear overall fold similarity of Macrodomain2 and Macrodomain3 to the experimentally determined SARS-CoV-2 Macrodomain1 (PDB code: 6WEY [36]) structure in terms of a core of beta strands (β1 through β5) with stereotypical interruption by α-helices. Using this, it was possible to determine the bounds of loop 1 (between β3 and the proximal downstream α-helix) and loop 2 (between β4 and the proximal downstream α-helix). Whereas the exact sequence alignment between these loop residues may not be precise, based on the fact that the sequences are so divergent, we are able to infer from those loop sequences that Macrodomain2 and Macrodomain3 lack the full repertoire of catalytic residues that would be expected to confer ADP-ribosylhydrolase enzymatic activity. Predicted structures, as well as experimentally determined structures for PARP14 Macrodomain1 bound to ADP-ribose (PDB code: 3Q6Z [37]) and SARS-CoV-2 Macrodomain1 (PDB code: 6WEY [36]) were displayed using PyMol (The PyMOL Molecular Graphics System, Version 2.1 Schrödinger, LLC. New York, NY, USA).

### 2.6. Plasmids and Constructs

For PARP10 overexpression, the coding sequence of human PARP10 (accession NP_116178.2) was cloned into the pcDNA5/FRT/TO backbone with an N-terminal mCherry, P2A linker, and 3×FLAG epitope tag. For macrodomain overexpression, codon-optimized sequences (see Supplemental File S4) were synthesized by Twist Biosciences (San Francisco, CA, USA) and cloned into pCMV-Twist with an N-terminal HA tag.

### 2.7. Cell Culture and Transient Transfection

Cell lines (HEK293T, obtained from ATCC (Manassas, VA, USA)), were routinely tested for mycoplasma infection using a PCR kit and kept at a low passage number. Cells were grown in complete media using DMEM (Gibco, Billings, MT, USA) with 10% FBS (Peak Serum, Wellington, CO, USA) and 1% Pen/Strep (Invitrogen, Carlsbad, CA, USA). Cells were seeded a day before transfection in a 24-well plate with 500 uL of media per well such that they would be at 60% confluent the following day for transfection. Cells were transfected using 500 ng of total plasmid DNA with 1.5 uL Transit-X2 transfection reagent (Mirus Bio, Madison, WI, USA) in 100 uL of OptiMEM (Invitrogen, Carlsbad, CA, USA) per well. In all assays, 100 ng of the plasmid expressed mCherry-P2A-3xFlag-PARP10. Except for the case shown in Figure S2, 250 ng of the HA-tagged macrodomain plasmid was used. In the case of Figure S2, either 25 ng, 100 ng, or 400 ng of HA-tagged macrodomain was transfected. In all transfections, the total amount of DNA added was supplemented to 500 ng with an empty cloning vector, pQCXIP (Clontech, Mountain View, CA, USA). Detection of ADP-ribosylation has been shown to be highly dependent on sample conditions [38], and we have observed that the edges of multiwell plates give less consistent signal than the middle of plates. As a result, only the central eight wells of any given plate were used for transfection.

### 2.8. Sample Preparation, Immunoblotting, and Antibodies

Cells that had been transfected with plasmids as described above were harvested 20 h post transfection. One hour prior to harvest, veliparib (VWR, Radnor, PA, USA), a selective PARP1/PARP2 inhibitor [39,40], was added to culture media to a final concentration of 1 μM to inhibit PARP1 activity as has been previously used [41]. At the time of harvest, media was aspirated, PBS was added to cells and aspirated, and then plates were frozen at −80 °C. After at least 1 h at −80 °C, plates were thawed on ice for 10 min and 75 uL of ADPr lysis buffer (50 mM Tris (pH 7.4), 150 mM NaCl, 1 mM MgCl₂, 1% triton-X-100, 1X protease inhibitor, 1 μM PDD00017273 (PARG inhibitor, Sigma-Aldrich, St. Louis, MO, USA)), 1 μM veliparib, 1 mM DTT) was added to each well. After a 10 min incubation on ice, lysates were transferred and centrifuged at 10,000× *g* at 4 °C for 5 min. The resulting supernatant was transferred to a new tube and 4× NuPAGE LDS sample buffer (Invitrogen) containing 5% β-mercaptoethanol (VWR) was added. Samples were boiled at 95 °C for 10 min and briefly centrifuged before being loaded onto a 4–12% Bis-Tris SDS-PAGE gel (Invitrogen) and run in 1X MOPS (Invitrogen) running buffer. Samples were then wet transferred onto nitrocellulose membrane and blocked with PBS-T containing 5% bovine serum albumin for 1 h. This was followed by incubation overnight at 4 °C with primary antibodies for mono/poly ADPr (anti-poly/mono-ADP-ribose antibody, E6F6A [42], Cell Signaling Technology, Danvers, MA, USA), anti-FLAG M2 (Sigma-Aldrich), anti-HA (Sigma-Aldrich), or anti-GAPDH (Cell Signaling Technology, Danvers, MA, USA). Membranes were then rinsed in PBS-T three times then incubated with the appropriate HRP-conjugated secondary antibodies (Fisher Scientific, Pittsburg, PA, USA). Membranes were then rinsed in PBS-T three times, and developed with SuperSignal West Pico PLUS Chemiluminescent Substrate (Fisher Scientific, Pittsburg, PA, USA), and imaged on a BioRad GelDoc (BioRad, Hercules, CA, USA).

## 3. Results

### 3.1. A Single Macrodomain in Human PARP9 and PARP14 Contains ADP-Ribosylhydrolase Activity

Among human PARP proteins, only PARP9, PARP14, and PARP15 contain a combination of macrodomains and a PARP domain (Figure 1A). To understand the distribution of macroPARPs within metazoans, we performed a phylogenetic analysis of homologs of PARP9, PARP14, and PARP15, characterizing them as either PARP9-like, PARP14-like, or PARP15-like based on their domain architecture and position within the protein phylogeny (Figure 1B). As previously observed, PARP15-like proteins only exist in mammalian species [8]. In contrast, we observed PARP9 homologs in jawed vertebrate species, including fish, reptiles, birds, and mammals, but lacking in the jawless sea lamprey, *Petromyzon marinus*, and non-vertebrate metazoans (Figure S1). PARP14 is the most broadly distributed in metazoans, with homologs in cnidarians (corals), spiralians (mollusks), and vertebrates, but noticeably absent in arthropods and nematodes (Figure 1B and Figure S1). From this, we conclude that the PARP14 domain structure of three tandem macrodomains and a PARP domain is the most ancestral form of macroPARP, with PARP9 and PARP15 arising in the vertebrate and mammalian lineages, respectively, as the result of partial duplication of PARP14. These date the existence of different macroPARPs in metazoans to >700 million years old for PARP14, ~500 million years old for PARP9, and ~100 million years old for PARP15 [43,44]. 

To further characterize the macrodomains present within metazoan macroPARPs, we extracted individual macrodomain sequences from each macroPARP and performed additional phylogenetic analyses. As shown in Figure 1C, the two macrodomains of PARP9 group phylogenetically with Macrodomain1 and Macrodomain2 of PARP14, respectively, whereas the two macrodomains of PARP15 correspond to Macrodomain2 and Macrodomain3 of PARP14, respectively. These data further support the model that PARP9 and PARP15 were partial duplications of the ancestral three macrodomain PARP14 architectures. 

**Figure 2:**
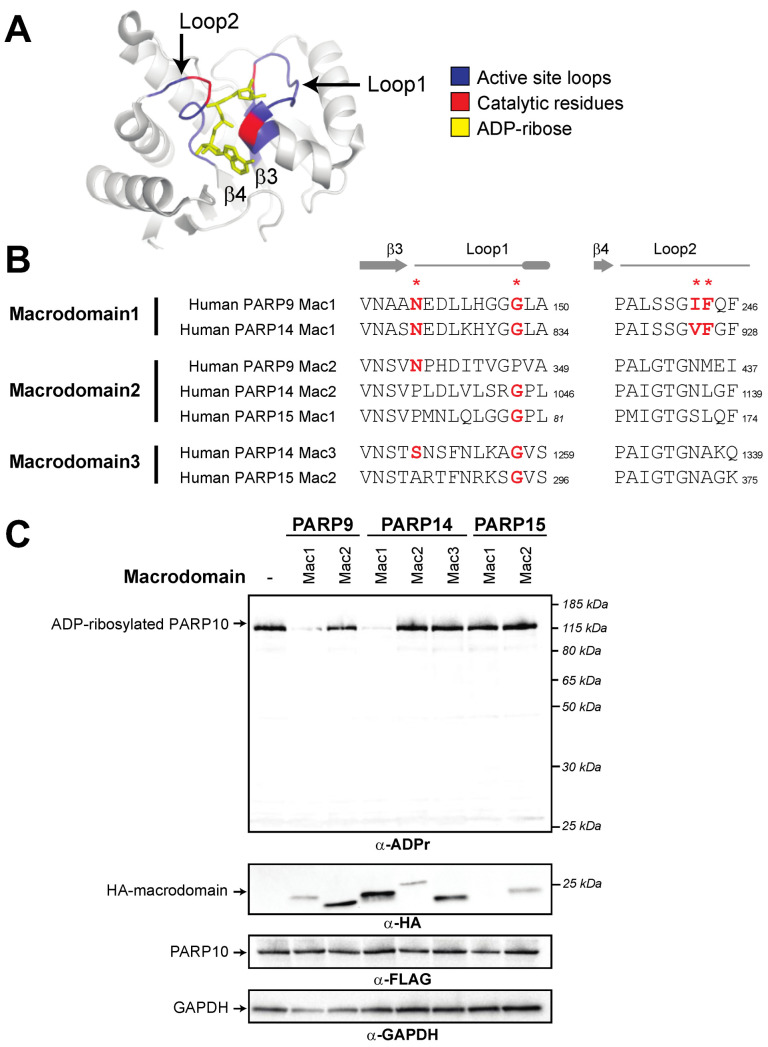
Presence of catalytic residues and enzymatic activity within individual macroPARP macrodomains. (**A**) Critical structural features and catalytic residues are mapped on the structure of PARP14 macrodomain1 solved in complex with ADP-ribose (PDB code: 3Q6Z [37]). Loop1 and Loop2, colored blue, are named in accordance with [46]. Important residues for ADP-ribosylhydrolase activity have been identified in several publications (see for example [18,45,46,47,48]) and are colored red. (**B**) Positions of catalytic residues (red asterisks) in Loop1 and Loop2 in human macroPARP macrodomains. Amino acids that are predicted to be compatible with enzymatic activity are shown in bold red. Residue number of the C-terminal residue in each motif is shown. (**C**) Enzymatic assay for ADP-ribosylhydrolase activity by transient overexpression of the indicated human macroPARP macrodomain with human PARP10 in human (HEK293T) cells. In the absence of any macrodomain, PARP10 (100 ng plasmid) is auto-ADP-ribosylated, resulting in a single band as detected by an anti-ADP-ribose antibody. A decrease in band intensity indicates that the indicated macrodomain (250 ng plasmid) is enzymatically active as an ADP-ribosylhydrolase. Anti-FLAG (PARP10) and anti-HA (macrodomain) blots are shown, as is an anti-GAPDH loading control. Expected positions of indicated proteins are shown, as are positions of molecular weight markers. Detailed information about the experimental protocol is found in the Section 2.

We next wished to ask which of the human macroPARP macrodomains display ADP-ribosylhydrolase activity. Several papers have described sequence characteristics that are important for host and viral macrodomain catalytic activity [18,45,46,47,48]. In particular, these analyses have focused on the importance of Asn/Ser and Gly residues flanking “loop 1” in the N-terminal region of the macrodomain and a hydrophobic (e.g., Ala, Thr, Ile, Val, or Leu) residue followed by an aromatic (e.g., Tyr or Phe) residue within “loop 2” in the C-terminal end of the protein (Figure 2A). Based on these features, we observed the presence of all of these key catalytic residues only in macrodomain1 of PARP9 and PARP14. As such, only Macrodomain1 would be expected to have catalytic activity, whereas the other macrodomains have sequence characteristics that would be predicted to inactivate ADP-ribosylhydrolase activity (Figure 2A).

To test these functional hypotheses, we expressed individual human macroPARP macrodomains with PARP10 and monitored auto-ADP-ribosylation of PARP10. We used ADP-riboslyation levels of PARP10 as a readout for ADP-ribosylhydrolase activity, since this is a commonly used substrate in the field [20,23,24,45,46,47]. In the absence of any macrodomain, PARP10 is robustly ADP-ribosylated as measured using an antibody that detects ADP-ribosylated proteins (Cell Signaling Technology anti-poly/mono-ADP-ribose antibody, E6F6A) [42]. As shown in Figure 2B, and confirming our bioinformatic predictions here and elsewhere [8], we only observed macrodomain ADP-ribosylhydrolase activity with Macrodomain1 of human PARP9 and human PARP14. Our results showing that Macrodomain1 of human PARP14 is an active ADP-ribosylhydrolase contrasts with a previous report that mouse PARP14 Macrodomain1 is enzymatically inactive [47]. The source of this discrepancy is unclear, but it should be noted that there are substantial differences in the methods used; whereas we assayed for activity from human cells in which macrodomains and PARP10 were overexpressed, the previous study used purified recombinant macrodomains and tested them against purified ADP-ribosylated PARP10 [47]. Beyond PARP9 and PARP14 Macrodomain1s, and again consistent with our bioinformatic predictions, other human macrodomains showed no obvious ADP-ribosylhydrolase activity, although the first macrodomain of PARP15 expresses poorly, so it is difficult to confirm a lack of enzymatic activity. Together, our bioinformatic and functional results indicate that two human macroPARP macrodomains are catalytically active, whereas the other five macrodomains found in human macroPARPs lack ADP-ribosylhydrolase activity.

### 3.2. Recurrent Loss of Macrodomain Enzymatic Activity in Mammalian PARP14s

We next sought to determine whether the existence of enzymatic activity within a given macroPARP macrodomain is conserved across species. We were particularly interested in this question as we had previously observed that all three macroPARPs are evolving under very strong positive selection in primates, with a large number of amino acid changes occurring in the macrodomains of each macroPARP [8]. We therefore considered the possibility that whereas human macroPARPs have catalytic activity in the Macrodomain1 of PARP14 and PARP9, other species may have a different constellation of macrodomains with enzymatic activity.

**Figure 3:**
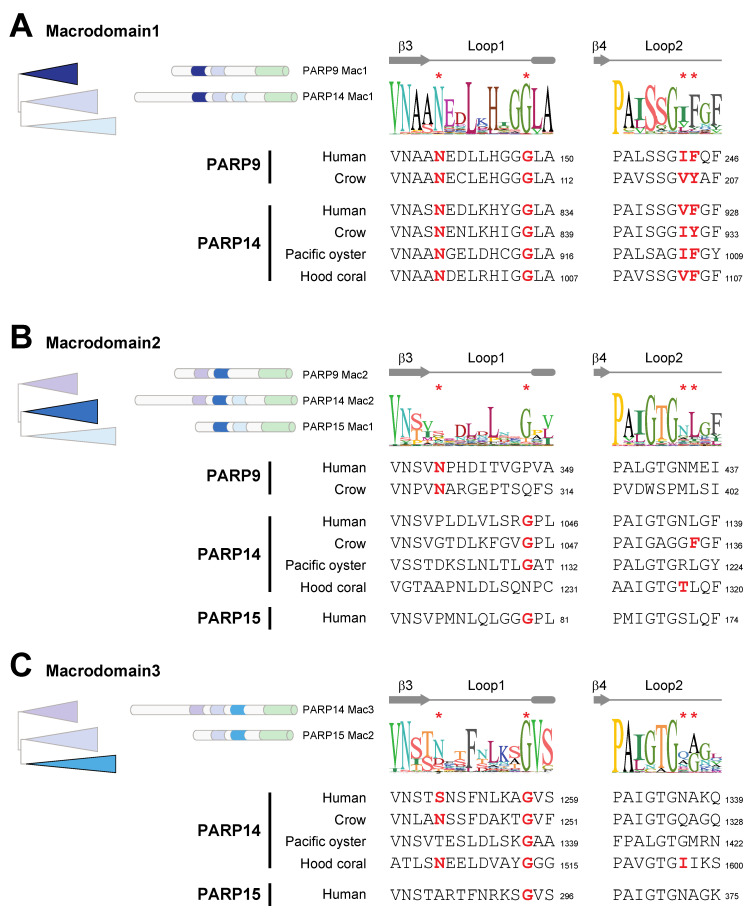
Catalytic residues are well conserved in macroPARP Macrodomain1 but not Macrodomain2 or Macrodomain3. (**A**) Cartoon of the phylogenetic position and protein position of Macrodomain1 as in Figure 1. A consensus logo of Loop1 and Loop2 across all analyzed Macrodomain1 sequences is shown, with critical residue positions indicated by red asterisks. Below are individual sequences from Macrodomain1s from vertebrates (human (*Homo sapiens)* and crow (*Corvus hawaiiensis)*), a spiralian (Pacific oyster, *Crassostrea gigas*) and a cnidarian (hood coral, *Stylophora pistillata*). Amino acids that are predicted to be compatible with enzymatic activity are shown in bold red. Residue number of the C-terminal residue in each motif is shown. (**B**) Same as A, except for Macrodomain2. (**C**) Same as A, except for Macrodomain3.

To first ask this question, we returned to our macrodomain alignments shown in Figure 1C and looked for conserved sequence features that might suggest gain or loss of enzymatic activity. Based on the sequence logos shown in Figure 3A–C, we predicted that only Macrodomain1, which is present in PARP9 and PARP14 but not PARP15, would be an enzymatically active ADP-ribosylhydrolase. Specifically, we found that sequence features that are required for catalytic activity are broadly conserved in Macrodomain1 from diverse species including cnidarians, spiralians, and vertebrates (Figure 3A). This includes our observation that all key catalytic residues are present in PARP14 Macrodomain1 from the hood coral, *Stylophora pistillata*, which is a cnidarian species and is therefore representative of a PARP14 macrodomain that diverged from mammalian PARP14 > 700 million years ago [43]. In contrast, using the same groups of species, we observed poor conservation of many of the key catalytic residues in Macrodomain2 (Figure 3B) and Macrodomain3 (Figure 3C). These results suggest that across metazoan macroPARPs, the ancestral state of PARP14 contained a catalytically active Macrodomain1, whereas Macrodomain2 and Macrodomain3 lacked catalytic activity.

**Figure 4:**
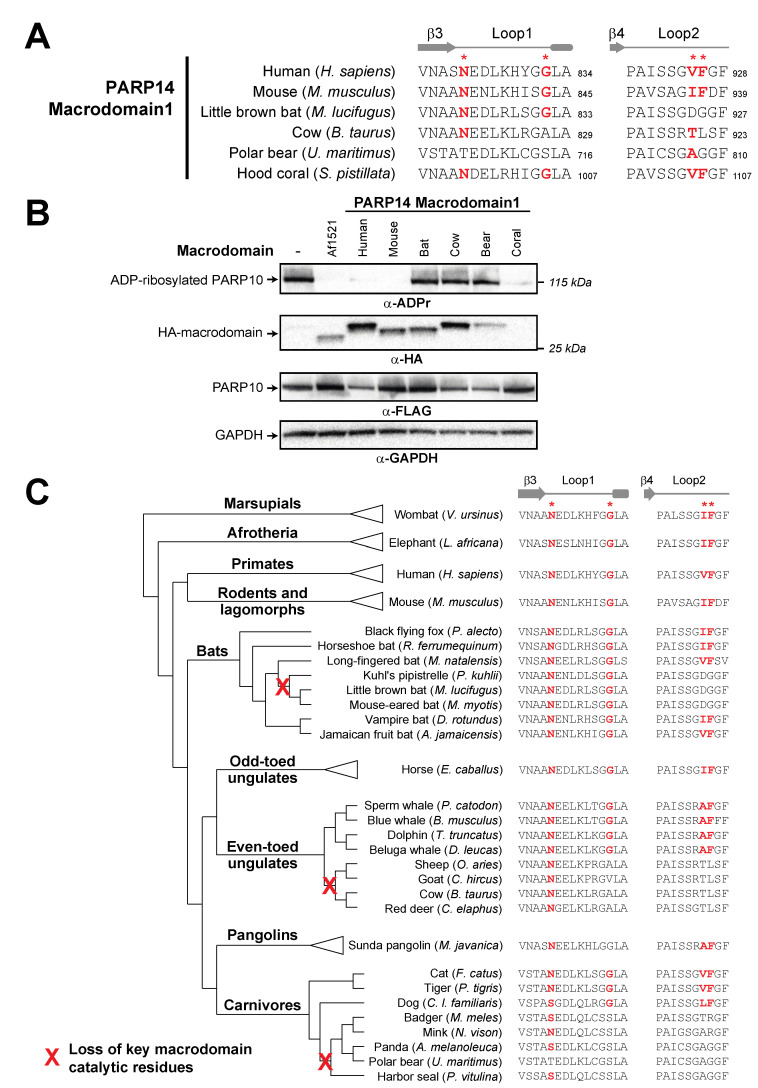
Recurrent loss of macrodomain enzymatic activity in mammalian PARP14. (**A**) Sequences of PARP14 Macrodomain1 from several metazoan species for Loop1 and Loop2. Critical residue positions indicated by red asterisks. Amino acids that are predicted to be compatible with enzymatic activity are shown in bold red. Residue number of the C-terminal residue in each motif is shown. (**B**) Enzymatic assay for ADP-ribosylhydrolase activity by transient overexpression of the indicated PARP14 Macrodomain1 with human PARP10 in human (HEK293T) cells as in Figure 2C. Expected positions of indicated proteins are shown, as are positions of molecular weight markers. As a positive control for ADP-ribosylhydrolase activity, the well-characterized macrodomain from *Archaeoglobus fulgidus* (Af1521) was included. Detailed information about the experimental protocol is found in the Section 2. (**C**) An expanded view of the phylogenetic tree for mammalian PARP14 from Figure 1B, with major mammalian clades and example species shown. To the right are sequences for each indicated species in Loop1 and Loop2, with red bold letters indicating presence of residues that are predicted to confer catalytic activity. Red “X’s” on the transformed phylogenetic tree indicate the inferred branch along which ADP-ribosylhydrolase activity was lost.

Interestingly, we did note that several species of mammals had mutations in the Macrodomain1 of PARP14 that disrupt critical residues for ADP-ribosylhydrolase activity. For instance, key residues have been mutated independently in P14 Macrodomain1 from little brown bat (*Myotis lucifugus*), cow (*Bos taurus*), and polar bear (*Ursus maritimus*) (Figure 4A). These data suggest that while Macrodomain1 has broadly retained enzymatic activity, several individual mammalian lineages have independently lost catalytic activity. 

To test the hypothesis that P14 Macrodomain1 from individual mammalian species has lost catalytic activity, we compared the activity of PARP14 Macrodomain1 from species we predicted would have catalytic activity to those that we predicted had lost catalytic activity (Figure 4B). As a positive control for ADP-ribosylhydrolase activity, we used the well-established macrodomain from the archael species *Archaeoglobus fulgidus* (Af1521) [49]. Consistent with our evolutionary model, we observed no catalytic activity for the PARP14 Macrodomain1 from cow, polar bear, and little brown bat. In contrast, PARP14 Macrodomain1 from humans and mice are enzymatically active, with robust ADP-ribosylhydrolase activity against PARP10 (Figure 4B,C). In addition, we observed that the cnidarian *S. pistillata* PARP14 Macrodomain1 also has robust catalytic activity, indicative of the ancient presence of ADP-ribosylhydrolase activity in macroPARP proteins (Figure 4B). 

To further characterize the evolutionary origins of the mutations to the key catalytic residues, we analyzed the PARP14 Macrodomain1 sequences from species closely related to those that had lost catalytic activity (Figure 4D). For instance, based on available bat PARP14 sequences, we infer that inactivating mutations that disrupt catalytic activity only arose in the vespertine microbats, including species in the *Myotis* and *Pipistrellus* genera. In contrast, other microbat species, including horseshoe bat (*Rhinolophus ferrumequinum*) and vampire bat (*Desmodus* rotundus), as well as megabats such as the black flying fox (*Pteroptus alecto*), retain all residues of the ancestral enzymatically active macrodomain. Likewise, within the even-toed ungulates (*Artiodactyla*), our data suggest that Macrodomain1 catalytic residues were disrupted in the ruminant lineage, including cow, sheep, goat, and deer, but are retained in cetaceans such as dolphins and whales. Finally, within carnivores, feline and canine species retain the indicated residues required for catalytic activity, whereas most other carnivores lack these critical residues. Together, our analyses shown in Figure 4B indicate at least three independent instances of loss of critical catalytic residues across the mammalian phylogeny. Based on estimates of divergence times of internal nodes in the mammalian phylogeny [50,51], all three of these independent losses of catalytic residues occurred between 25 and 65 million years ago during the mammalian diversification that followed the Cretaceous–Paleogene (KPg) mass extinction. 

### 3.3. Tandem Macrodomain Orientation Is Shared between MacroPARPs and Coronaviruses

**Figure 5:**
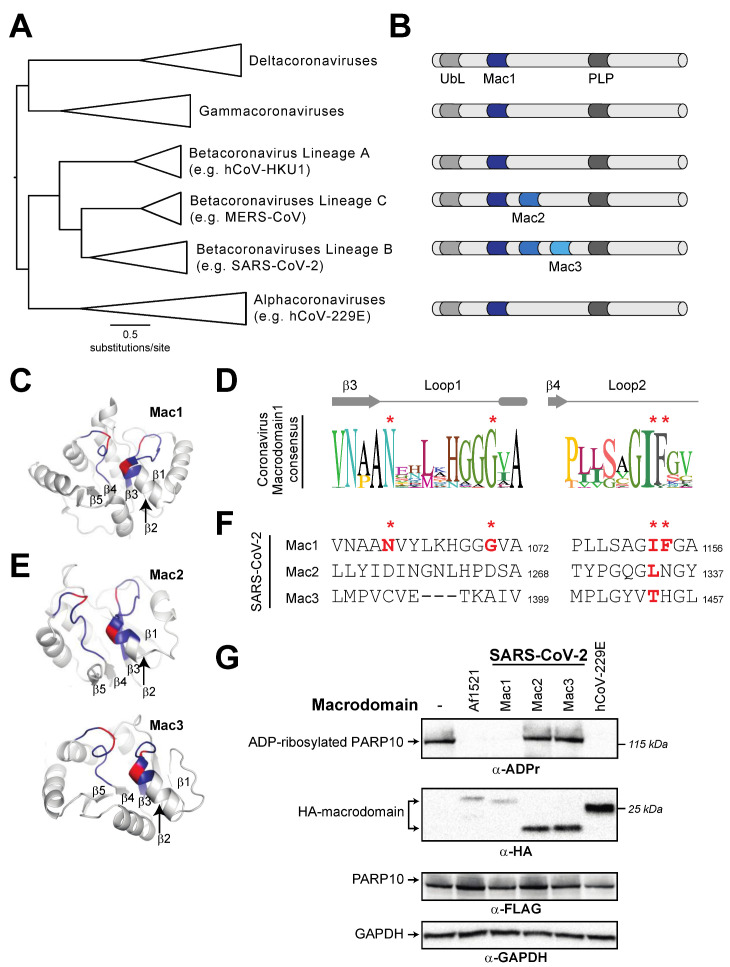
Distribution of active and inactive macrodomains in coronaviruses. (**A**) Phylogenetic tree of nsP3 proteins from diverse coronaviruses. Major coronavirus clades are shown. (**B**) Domain cartoon of nsP3 proteins from the indicated coronavirus clades. Flanking nsP3 macrodomains are a ubiquitin-like (UbL) domain and a papain-like protease (PLP). The number of macrodomains found in each protein is shown. For clarity, other domains are not shown. (**C**) Critical structural features and catalytic residues are mapped on the structure of the SARS-CoV-2 nsP3 macrodomain1 (PDB code: 6WEY [36]). Loop1 and Loop2, colored blue, and important residues for ADP-ribosylhydrolase activity, colored red, as indicated as in Figure 2A. The first five beta-strands in the structure are also labeled. (**D**) Consensus logo of Loop1 and Loop2 across all analyzed coronavirus Macrodomain1 sequences. Key residue positions are marked by red asterisks. (**E**) Structural models for SARS-CoV-2 nsP3 Macrodomain2 and Macrodomain3 were predicted using AlphaFold2 via the ColabFold package [35]. Loops, positions of important residues, and beta-strands are marked as in part C. (**F**) Sequences of Loop1 and Loop2 from the indicated SARS-CoV-2 macrodomains. Although there is little sequence similarity to other viral or host macrodomains, the sequences of Loop1 and Loop2 in Macrodomain2 and Macrodomain3 were identified using the structural models shown in panel (**E**) (see Materials and Methods for additional explanation). Red bold letters indicate presence of residues that are predicted to confer catalytic activity. Residue number of the C-terminal residue in each motif relative to the start of the viral ORF1ab polyprotein is shown. (**G**) Enzymatic assay for ADP-ribosylhydrolase activity by transient overexpression of the indicated coronavirus macrodomain with human PARP10 in human (HEK293T) cells as in Figure 2C. Expected positions of indicated proteins are shown, as are positions of molecular weight markers. Detailed information about the experimental protocol is found in the Section 2.

Having analyzed macrodomain activity in host macroPARPs, we next turned our attention to viral macrodomains. Coronaviruses have a conserved macrodomain that has been the target of substantial interest, as it is required for antagonizing the host immune response [12,17,20,21,22]. Interestingly, several coronaviruses, including SARS-CoV-2, encode tandem macrodomains, as is seen in host macroPARPs (Figure 5A,B). We therefore wished to determine whether the catalytic residues that are required for ADP-ribosylhydrolase activity are conserved in one or all coronavirus macrodomains. As with macroPARPs, we found that Macrodomain1 contains conserved residues that are predicted to be consistent with catalytic activity (Figure 5C,D), although in this case, we observed no cases in which the catalytic residues were mutated in any coronavirus. However, it was difficult to reliably align Macrodomain2 and Macrodomain3 to Macrodomain1 based on primary sequence alone. As a result, we performed structural predictions using AlphaFold [35] to identify residues in positions that are analogous to Loop1 and Loop2 in Macrodomain1 (Figure 5C,E). These structure-based homology models allowed us to predict the absence of catalytic residues in the SARS-CoV-2 Macrodomain2 and Macrodomain3 (Figure 5F).

We next wished to test the hypothesis that, like macroPARPs, only the first macrodomain of the SARS-CoV-2 tandem macrodomains is enzymatically active. We therefore cloned and expressed individual macrodomains from SARS-CoV-2 as an example of a three-macrodomain viral protein and from hCoV-229E as an example of a one-macrodomain viral protein. As with PARP14 and PARP9, we observed that Macrodomain1 of each virus had measurable ADP-ribosylhydrolase activity, which is consistent with several previous studies [12,18,20,21] (Figure 5G). In contrast, we observed no activity from Macrodomain2 or Macrodomain3 from SARS-CoV-2, consistent with the absence of residues required for catalytic activity and with prior observations that Macrodomain2 and Macrodomain3 of SARS-CoV specifically bind nucleic acids [52].

### 3.4. Recurrent Loss of Macrodomain Activity in Alphaviruses

We finally wished to analyze the macrodomain activity within the alphavirus genus of *Togaviridae*. Alphaviruses encode a single macrodomain within the nsP3 protein that has important roles in tissue tropism, viral replication and virulence [23,24,25,26]. Indeed, previous macrodomain mutations have been shown to prevent replication in both mammalian and mosquito cells [24]. Based on this functional importance, as well as the observed conservation of macrodomains across diverse coronaviruses (Figure 5D and [18]), we therefore expected strong conservation of macrodomain sequences and catalytic activity within the alphaviruses. 

**Figure 6:**
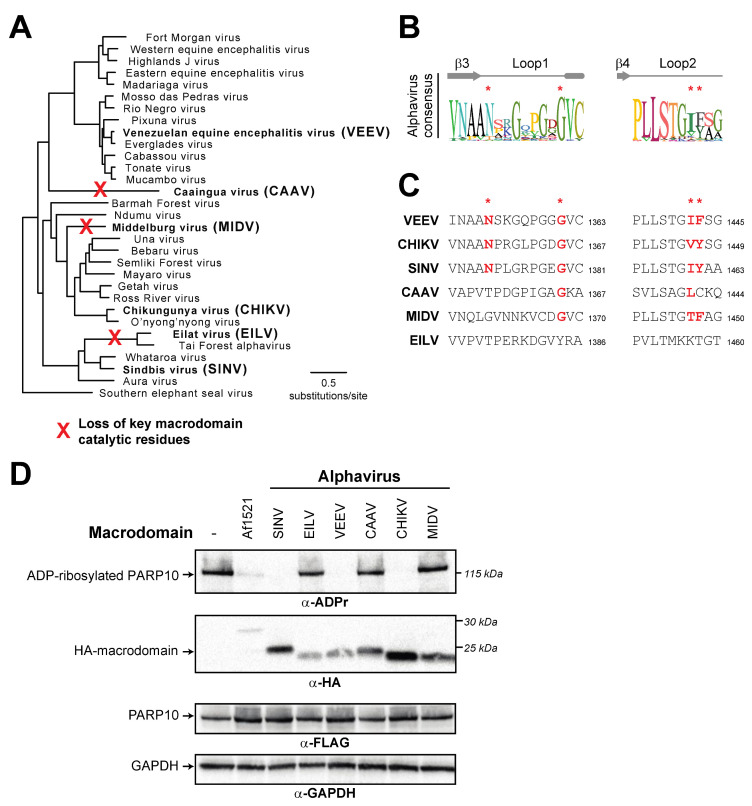
Recurrent loss of macrodomain enzymatic activity in alphaviruses. (**A**) Phylogenetic tree of nonstructural polyproteins from diverse alphaviruses. Species in bold are those that are shown in panels (**C**,**D**). Red “X’s” indicate the inferred branch along which ADP-ribosylhydrolase activity was lost based on data shown in panel (**C**,**D**). (**B**) Consensus logo of Loop1 and Loop2 across all analyzed alphavirus macrodomain sequences. Key residue positions are marked by red asterisks. (**C**) Sequences of Loop1 and Loop2 from the indicated alphavirus macrodomains. Red bold letters indicate presence of residues that are predicted to confer catalytic activity. Residue number of the C-terminal residue in each motif relative to the start of the viral nsP1-4 polyprotein is shown. (**D**) Enzymatic assay for ADP-ribosylhydrolase activity by transient overexpression of the indicated alphavirus macrodomain with human PARP10 in human (HEK293T) cells as in Figure 2C. Expected positions of indicated proteins are shown, as are positions of molecular weight markers. Detailed information about the experimental protocol is found in the Section 2.

We first generated a phylogenetic tree of nonstructural polyproteins from diverse alphaviruses (Figure 6A). We then extracted macrodomain sequences from these viruses. Similar to the macroPARP Macrodomain1 alignment (Figure 3A), we observed a consensus sequence that contained residues shown to be important for catalysis, but also observed that these residues were not perfectly conserved (Figure 6B). We therefore investigated whether any alphaviruses might lack residues important for ADP-ribosylhydrolase activity. Consistent with previous observations [18], we noted that the insect-specific alphaviruses, including Eilat virus (EILV) [53] and Tai Forest virus [54], lack residues expected to confer enzymatic activity (Figure 6C). More surprisingly, we also found two separate additional cases of alphavirus macrodomains that lacked one or more of the catalytic residues. The first occurs in Middelburg virus (MIDV), a virus which has been isolated from cerebrospinal fluid and blood from humans [55] and is associated with severe disease in horses [56]. Importantly, phylogenetic analyses indicate that MIDV is more closely related to several human alphaviruses than it is to the insect-specific alphaviruses (Figure 6A), suggesting this was an independent loss of macrodomain catalytic activity. Finally, we observed another loss of catalytic residues in a recently discovered virus known as Caaingua virus (CAAV), which was isolated from mosquitoes but could be cultured in human mononuclear cells [57]. Consistent with prior phylogenetic analyses [57], we found Caaingua groups with other vertebrate-infecting viruses, rather than with the insect-specific viruses (Figure 6A). 

To test the hypothesis that macrodomains from EILV, MIDV, and CAAV are enzymatically inactive, we cloned and expressed each macrodomain in the presence of human PARP10. In addition to the panel of alphavirus macrodomains we predicted would be inactive, we also tested several diverse alphavirus macrodomains that we predicted would be active, including those from Sindbis virus (SINV), chikungunya virus (CHIKV), and Venezuelan equine encephalitis virus (VEEV). Consistent with our predictions from sequence data, we observed no ADP-ribosylhydrolase activity against PARP10 with EILV, MIDV, and CAAV, whereas we observed robust activity with the other alphavirus macrodomains (Figure 6D). These data indicate that ADP-ribosylhydrolase activity is not absolutely essential for a macrodomain in a vertebrate-infecting alphavirus. In addition, when placed in the context of our phylogenetic analyses (Figure 6C), these data suggest that, like PARP14, macrodomain activity has been independently lost several times in the alphaviruses.

## 4. Discussion

Addition, recognition, and removal of ADP-ribosylation has emerged as an important battleground between the host antiviral immune response and viruses. Although the molecular targets of ADP-ribosylation, and the mechanistic consequences of that ADP-ribosylation, are mostly uncharacterized, there is clear function of host PARPs in the antiviral immune response and a clear role of viral macrodomains in antagonizing the host immune response [17,58,59,60,61]. Our evolutionary and functional data suggest that an important consideration is the degree to which the ADP-ribosylhydrolase activity encoded by some macrodomains is conserved amongst host antiviral macroPARPs and is conserved amongst viral macrodomains. 

In addition to ADP-ribosylhydrolase activity, macrodomains have been observed to have several other functions. In particular, some macrodomains that lack the ability to remove (‘erase”) ADP-ribosylation still retain the ability to recognize (‘read”) ADP-ribosylated proteins [5,6,7]. In most of the cases we describe, individual substitutions in catalytic residues would likely still retain this “reader” function, potentially allowing these proteins to still function in some aspects of ADP-ribose biology. In addition, macrodomains have been shown to function in processes not directly related to protein ADP-ribosylation, including binding nucleic acids and catalyzing degradation of a product of tRNA splicing [5,6,7], and it is unknown whether the enzymatically inactive macrodomains may participate in these functions. By sampling macrodomain diversity found throughout metazoan macroPARPs and viruses, additional insights into the many functions of macrodomains may emerge.

Although the recurrent loss of enzymatic activity in an important host–virus battleground is seemingly paradoxical, it is reminiscent of the observation that two interferon-stimulated antiviral PARPs lack the ADP-ribosyltransferase activity that all of the other mammalian PARPs display. PARP13, also known as zinc-finger antiviral protein (ZAP), lacks ADP-ribosyltransferase activity but is a potent antiviral factor against a wide range of viruses, including retroviruses and alphaviruses [62]. In addition, PARP9 is a macroPARP that can potentiate the IFN response [16], but lacks the canonical activity of other PARPs. Previously characterized as a catalytically inactive ADP-ribosyltransferase like PARP13 due to a lack of conserved catalytic residues [3], PARP9 has been implicated in specific ADP-ribosylation of ubiquitin when in complex with its E3-ubiquitin ligase binding partner, DTX3L [63]. Regardless of potential residual enzymatic activity in PARP9, it is striking that the only two PARPs that lack canonical ADP-ribosyltransferase activity are also upregulated by the antiviral cytokine IFN, have antiviral function, and are evolving under strong positive selection indicative of a host–virus genetic conflict [8]. Whether the loss of enzymatic activity in those antiviral PARPs, or whether the loss of macrodomain enzymatic activity in the antiviral host protein PARP14, is adaptive or confers some additional function to these proteins remains to be determined. 

In addition, the loss of macrodomain activity in several alphavirus lineages is surprising. Whereas the loss of macrodomain activity in insect-specific alphaviruses may be rationalized by the observation that insects lack most PARP proteins found in vertebrates, the loss of catalytic activity from Caaingua and especially Middelburg viruses is more difficult to reconcile. It will be interesting to determine how these viruses can infect human cells while lacking ADP-ribosylhydrolase activity that has been shown to be critical for other alphaviruses.

In sum, our results highlight an unexpected but recurrent loss of enzymatic activity in host and viral macrodomains in a way that will fundamentally affect their interactions with protein ADP-ribosylation. Such observations go against the assumption that macrodomain activity will be broadly conserved, especially in viruses. These results indicate that there remain many aspects of ADP-ribosylation, especially at the interface between the host antiviral immune response and viruses, that remain to be fully understood. 

## Data Availability

All data are contained within the article or supplementary material. All sequences used for analysis are available in the NCBI protein database (https://www.ncbi.nlm.nih.gov/protein/, accessed on 1 January 2023) using the accession numbers shown in the supplementary material.

## Supplementary Materials

The following supporting information can be downloaded at: https://www.mdpi.com/article/10.3390/pathogens12050674/s1, File S1: Accession numbers and species names of metazoan macroPARPs used for phylogenetic analyses; File S2: Accession numbers and species names of coronavirus macrodomain-containing proteins used for phylogenetic analyses; File S3: Accession numbers and species names of alphavirus nsP3 macrodomain-containing proteins used for phylogenetic analyses; File S4: Macrodomain sequences used for functional analyses; Figure S1: Phylogenomic distribution of macroPARPs in metazoans; Figure S2: Dose response of human and bat (M. lucifugus) PARP14.
