# Supplementary material for: Recurrent Loss of Macrodomain Activity in Host Immunity and Viral Proteins
Source: Pathogens. 2023 May 3;12(5):674. doi: 10.3390/pathogens12050674 (PMC10221186; doi:10.3390/pathogens12050674)
Supplement: Supplementary file 1 [file pathogens-12-00674-s001.zip › Supplemental figures S1 and S2.pdf]

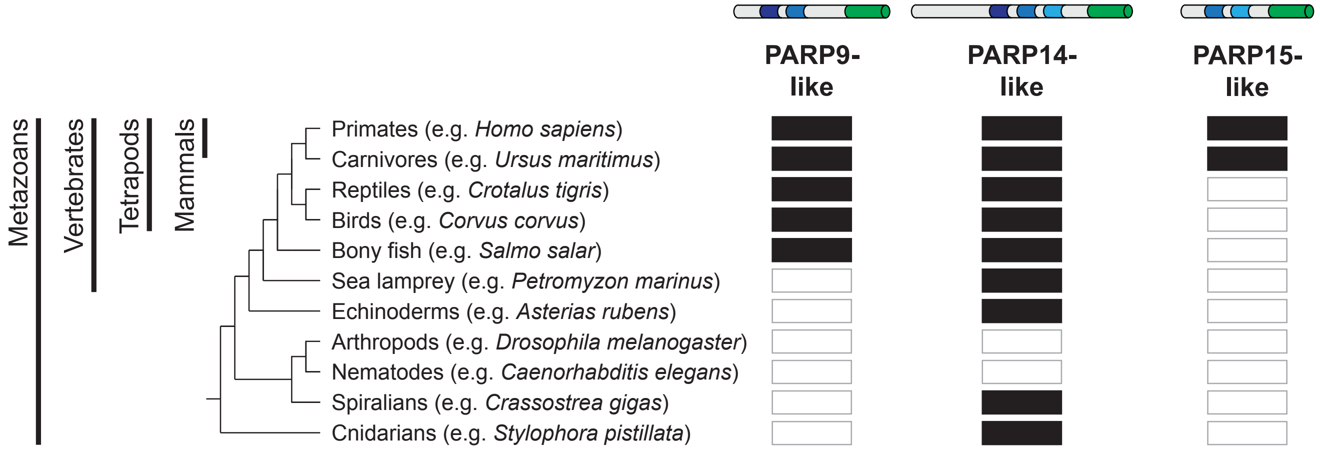

Figure S1. Phylogenomic distribution of macroPARPs in metazoans. Presence of a PARP9-like, PARP14-like, or PARP15-like protein in the genome of the indicated species is shown. Data are based on presence of proteins within the phylogenetic tree shown in Figure 1B. Lack of macroPARPs in the indicated genome was validated by absence of an ortholog using BLASTP and HMMER searches with an e-value cutoff of 0.05 (see Materials and Methods). Absence of all macroPARPs in indicated arthropods and nematodes was reported previously in Otto et al., *BMC Genomics*, 2005 (reference 31 in main manuscript)

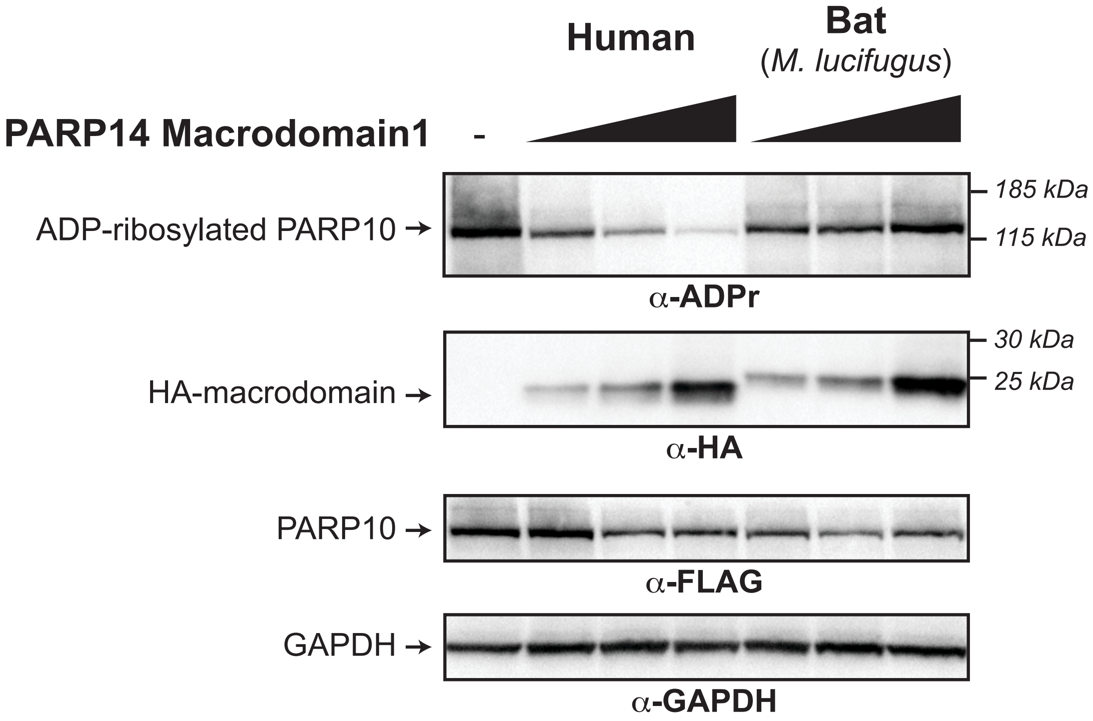

Figure S2. Dose response of human and bat (*M.* lucifugus) PARP14. Assays were performed as in Figure 2C, except using increasing amounts of HA-tagged macrodomain. Plasmid concentrations used were 25ng, 100ng, and 400ng. Detailed information about the experimental protocol is found in the Materials and Methods section.
